# Distinct cortico-muscular coupling between step and stance leg during reactive stepping responses

**DOI:** 10.3389/fneur.2023.1124773

**Published:** 2023-03-14

**Authors:** Mitchel Stokkermans, Teodoro Solis-Escalante, Michael X. Cohen, Vivian Weerdesteyn

**Affiliations:** ^1^Department of Rehabilitation, Radboud University Medical Center for Medical Neuroscience, Nijmegen, Netherlands; ^2^Department of Synchronisation in Neural Systems, Donders Institute for Brain Cognition and Behavior, Nijmegen, Netherlands; ^3^Sint Maartenskliniek Research, Nijmegen, Netherlands

**Keywords:** balance control, corticomuscular coupling, EEG, reactive stepping, motor control

## Abstract

Balance recovery often relies on successful stepping responses, which presumably require precise and rapid interactions between the cerebral cortex and the leg muscles. Yet, little is known about how cortico-muscular coupling (CMC) supports the execution of reactive stepping. We conducted an exploratory analysis investigating time-dependent CMC with specific leg muscles in a reactive stepping task. We analyzed high density EEG, EMG, and kinematics of 18 healthy young participants while exposing them to balance perturbations at different intensities, in the forward and backward directions. Participants were instructed to maintain their feet in place, unless stepping was unavoidable. Muscle-specific Granger causality analysis was conducted on single step- and stance-leg muscles over 13 EEG electrodes with a midfrontal scalp distribution. Time-frequency Granger causality analysis was used to identify CMC from cortex to muscles around perturbation onset, foot-off and foot strike events. We hypothesized that CMC would increase compared to baseline. In addition, we expected to observe different CMC between step and stance leg because of their functional role during the step response. In particular, we expected that CMC would be most evident for the agonist muscles while stepping, and that CMC would precede upregulation in EMG activity in these muscles. We observed distinct Granger gain dynamics over theta, alpha, beta, and low/high-gamma frequencies during the reactive balance response for all leg muscles in each step direction. Interestingly, between-leg differences in Granger gain were almost exclusively observed following the divergence of EMG activity. Our results demonstrate cortical involvement in the reactive balance response and provide insights into its temporal and spectral characteristics. Overall, our findings suggest that higher levels of CMC do not facilitate leg-specific EMG activity. Our work is relevant for clinical populations with impaired balance control, where CMC analysis may elucidate the underlying pathophysiological mechanisms.

## 1. Introduction

Performing daily activities (e.g., standing or walking) constantly challenges postural balance. With different postures and activities, continuous adaptation through contraction and relaxation of specific muscles allows the control of balance and the execution of corrective steps whenever these are needed. In the past decade, many studies using mobile EEG have provided evidence that the cortex plays an important role in postural control (1–3). This notion is in line with studies investigating postural control in people with cortical lesions (e.g., stroke), which reported deficient recruitment of the muscles involved in perturbation-evoked responses, including delayed onset latencies, lower response amplitudes, and aberrant coordination patterns across muscles (4, 5).

Postural perturbations are known to elicit several event-related potentials, suggesting cortical involvement in the ensuing balance recovery responses. The initial P1 response (30–90 ms) is thought to represent proprioceptive sensory afferents (6), followed by the N1 (90–200 ms), which is suggested to reflect monitoring of postural stability (1, 7–11). The N1 perturbation-related response is accompanied by a transient power increase of the theta (3–8 Hz) rhythm (12). Interestingly, the N1 and theta rhythm have been shown to predict the response outcome to a balance perturbation with stronger cortical dynamics for stepping responses compared to feet-in-place responses at similar perturbation intensities (12). Yet, little is known about the temporal evolution of cortical interaction with specific leg muscles that contribute to the generation of the balance correcting response (either directly or indirectly through cortico-cortical coupling with the motor areas).

Cortico-muscular coupling (CMC) is a powerful analysis tool to investigate functional connectivity between the cerebral cortex and muscles across the body. CMC is known to take place with the Tibialis Anterior (TA) and Soleus (SO) muscles at beta (15–25 Hz) frequencies during isometric contraction and gamma (40–80 Hz) frequencies during isotonic contraction (13, 14). In addition, several studies reported beta frequency coupling with the medial Gastrocnemius (MG) and TA muscles during cyclic ankle movements (15). These findings suggest that multiple cortical frequency bands may couple to similar muscles during different muscle exercises. Until today, only a few studies have investigated CMC of dynamic human behavior related to postural control and gait. Moreover, studies investigating gait reported an increase in theta, alpha, and beta band CMC after foot strike with TA, Vastus Medialis (VM), Biceps Femoris (BF), Peroneus longus (PL), MG and SO muscles (16–18). Cortical involvement in the balance response is thought to occur during later phases of the balance recovery response (19, 20), which suggests that reactive step responses may coincide with strong CMC time locked to perturbation onset. Only few studies investigated CMC during walking and standing balance (17, 21). Muscle synergies showed strong coherence with cortical activity over the Piper rhythm (~40 Hz) during lateral balance perturbation of unipedal stance that led to feet-in-place responses (21). In addition, strong theta and alpha (8–13 Hz) band CMC dynamics were observed from cortex to muscle with MG, TA and PL muscles during physical standing perturbations leading to feet-in-place responses compared to visual perturbations while standing (17). This indicates that physical perturbations require more cortical control. Yet, their experimental setup only focused on the theta and alpha frequency bands averaged over a window of 1 s, thus lacking information on the temporal evolution of cortical interaction with the muscles during the balance response. In addition, beta and gamma frequency bands are motor task related rhythms, which may also be involved in the recruitment of balance responses.

The aim of this study was to investigate CMC from cortex to muscle through spectral Granger causality coupled to key events of the stepping response in balance. We hypothesized that CMC would occur over multiple frequency bands during stepping responses. In addition, we expected CMC to differ between the step and stance leg according to their differential muscle recruitment patterns inherent in executing the step response. In particular, we expected that stronger CMC would be most evident for the muscles involved in generating the greatest biomechanical contribution for the stepping movement (depending on the step and stance leg), and that an increase in CMC would precede changes in EMG activity in these muscles relative to foot off and foot strike event. Therefore, we conducted separate CMC analysis time-locked to perturbation onset, foot-off and foot strike event.

## 2. Materials and methods

Twenty young healthy adults participated in this study. We analyzed a total of 18 datasets (8 female; age mean 23.9 years, sd 3.6 years) due to technical issues in two other datasets. All participants received ample information about the experiment and signed an informed consent document prior to the start of the experiment. Participants were financially compensated after completion of the study. None of the participants had a history of neuromuscular disease or any other impairment that could affect their performance in the experiment. The experimental procedure was approved by the Research Ethics Committee of the Radboud University Medical Center (Nijmegen, The Netherlands; Dossier 2018-4970). The experiments were conducted in line with the Declaration of Helsinki.

### 2.1. Experimental paradigm

**Figure 1:**
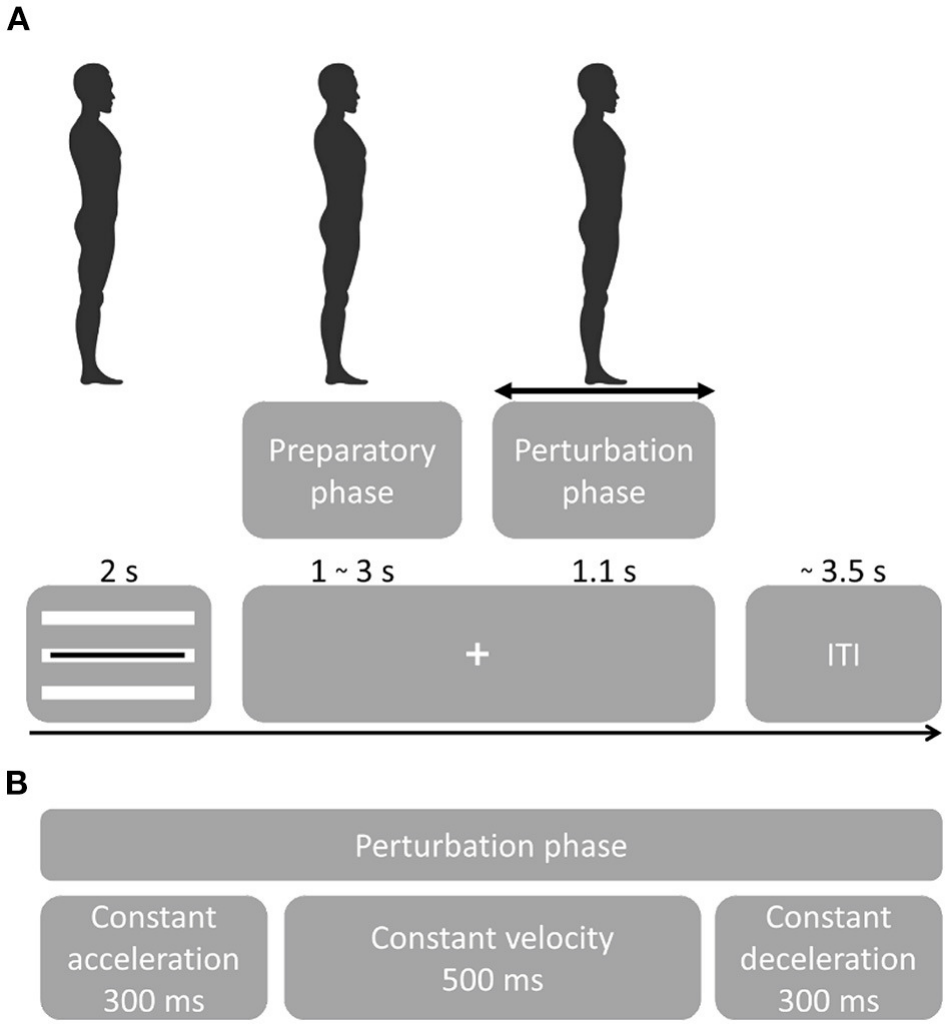
Experimental procedure. **(A)** Participants were instructed to maintain a straight posture during the sequence of perturbations. Perturbation onset times were randomized as well as direction and acceleration. A visual cue tracking the participant's posture was presented for 2 s, followed by a fixation cross. Platform onset was randomized from 1–3 s followed by a perturbation that lasted 1.1 s. Following a perturbation, the platform returned to the initial position. At platform return, the visual feedback of the leaning posture was presented. Feedback for the leaning posture [important for the experimental setup in (22)] was presented through three white bars representing forward leaning (top bar), neutral stance (middle bar) and backward leaning (bottom bar). The black bar presented on top of the white bars, indicated the participant's real-time leaning angle. Participants were instructed to maintain the black bar on the white bar corresponding to the instructed leaning posture. The initial leaning posture had to be maintained while the fixation cross was presented ensuring that postural stability was controlled at platform perturbation. **(B)** Platform perturbation profiles.

Data used in this study were derived from a protocol to investigate theta power modulations related to balance monitoring by imposing leaning angles prior to perturbation (22) (Figure 1A). Participants were familiarized with the balance platform through a series of 28 forward and backward perturbations with increasing acceleration, delivered by the Radboud Falls Simulator (2, 12, 23, 24). Participants stood barefoot on the movable platform with their feet at shoulder width and their arms crossed in front of the body and had to maintain three different initial leaning postures prior to a balance perturbation. At the beginning of each sequence, the participants were instructed about which leaning posture to maintain throughout the series of perturbations. Participants were instructed and encouraged to respond with feet-in-place responses following balance perturbations. A real-time 3D-motion data stream monitored the participants' posture and performance (Vicon motion systems, United Kingdom), such as maintaining leaning angle, excessive knee flexion and changes in leg weight bearing (which may indicate whether specific strategies to counteract balance perturbations were used).

Balance perturbation profiles consisted of 300 ms platform acceleration, 500 ms constant velocity and 300 ms deceleration (Figure 1B). Platform accelerations were randomized and ranged from 0.25 to 1.9 m/s^2^ with a higher resolution at lower accelerations in both forward and backward sway direction (0.25, 0.4, 0.7, 1.0, 1.3, 1.6, 1.9 m/s^2^). The initial experiment contained 15 sequences with 29 balance perturbations each (435 total). The first perturbation of a sequence always consisted of a low-intensity dummy trial and was not included in the analyses. For the analysis of the present study, we only considered the neutral stance conditions with forward and backward perturbation directions (i.e., 140 trials per participant).

Participants took a small break of 5 min after three consecutive sequences and were seated on a chair to prevent fatigue. After nine perturbation sequences, participants were given a 20-min resting break. The active experiment time was 2.5 h and the preparation time was 2.5 h, the complete lab visit lasted a maximum of 6 h (including resting breaks).

### 2.2. Data acquisition

We recorded high-density EEG using a cap with 126 Ag-AgCl electrodes (WaveGuard, ANT Neuro, The Netherlands). The electrodes were fixed in the cap and distributed across the scalp according to the five percent electrode system (25). The EEG data were referenced to the common average during acquisition. The ground electrode was placed on the left mastoid. A biosignal amplifier (REFA System, TMSi, The Netherlands) recorded the EEG at 2,048 Hz without any filters, except for a hardware low-pass filter at 552 Hz. To monitor physiological activity that could present artifacts in the EEG, we also recorded electrical activity of the left eye in the vertical and horizontal direction (electrooculogram, EOG) using adhesive Ag-AgCl electrodes. The EOG was recorded from electrodes placed slightly under the left eye (vertical eye movement) and at the outer canthus of the left eye (horizontal eye movement).

**Figure 2:**
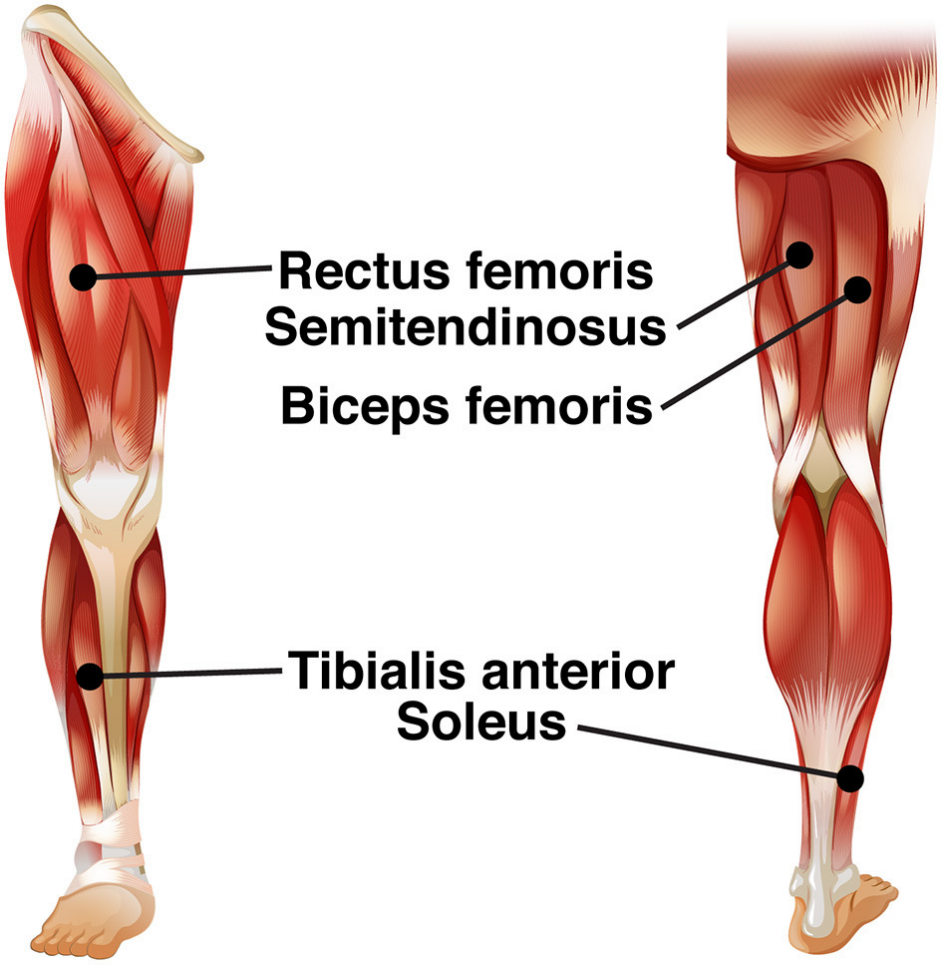
EMG electrode leads.

We recorded electrical activity bilaterally from five leg muscles [see Figure 2; soleus (SO), tibialis anterior (TA), rectus femoris (RF), biceps femoris (BF), semitendinosus (ST)], using surface EMG electrodes (Mini Wave, Cometa systems, Italy). Muscle sites were shaved to remove hair, and the skin was scrubbed with skin preparation gel (Nuprep, MedCat) to improve skin conduction and cleaned with alcohol. The EMG amplifier (Wave plus wireless, Cometa, Italy) recorded muscle activity at 2,000 Hz. The EMG signal of the muscles was carefully checked before the start of the experimental paradigm.

Body movement was recorded using an 8-camera 3D motion analysis system (Vicon motion systems, United Kingdom) at a sample rate of 100 Hz. For this purpose, a total of 23 reflective markers (PlugInGait Full-body AI model excluding the head and arm markers; Vicon Nexus software 2.7.1) were attached to anatomical landmarks on the participants' body.

Ground reaction forces were recorded from two force plates (AMTI Custom 6 axis composite force platform, USA; size: 60 × 180 cm each; sampling rate: 2,000 Hz) embedded in the moveable platform. Trials were recorded from−2 to +5 s relative to the platform perturbation. Synchronization triggers were generated by the platform controller and recorded for *post-hoc* alignment of EMG, EEG and motion data.

### 2.3. EMG processing

The EMG signal was preprocessed in MATLAB using low-pass filtering with the ‘filtfilt.m' function (125 Hz low-pass filtered 5th order Butterworth IIR filters, zero-phase shift), and downsampling to 250 Hz. EMG was separately preprocessed for EMG envelope visualization and Granger causality.

For EMG envelope visualization, the data were band-pass filtered (20–120 Hz band-pass 5th order Butterworth IIR filters, zero-phase shift), full-wave rectified, low-pass filtered (40 Hz low-pass 5th order Butterworth IIR filters, zero-phase shift), and normalized per muscle per subject to the maximum muscle activation at 0.7 m/s^2^ for feet-in-place responses. This normalization allowed us to evaluate the difference in activation between step and stance legs during stepping trials since both legs contributed equally in the feet-in-place trials. The acceleration of 0.7 m/s^2^ was the maximum platform acceleration where all participants were able to respond with feet-in-place in either direction.

**Figure 3:**
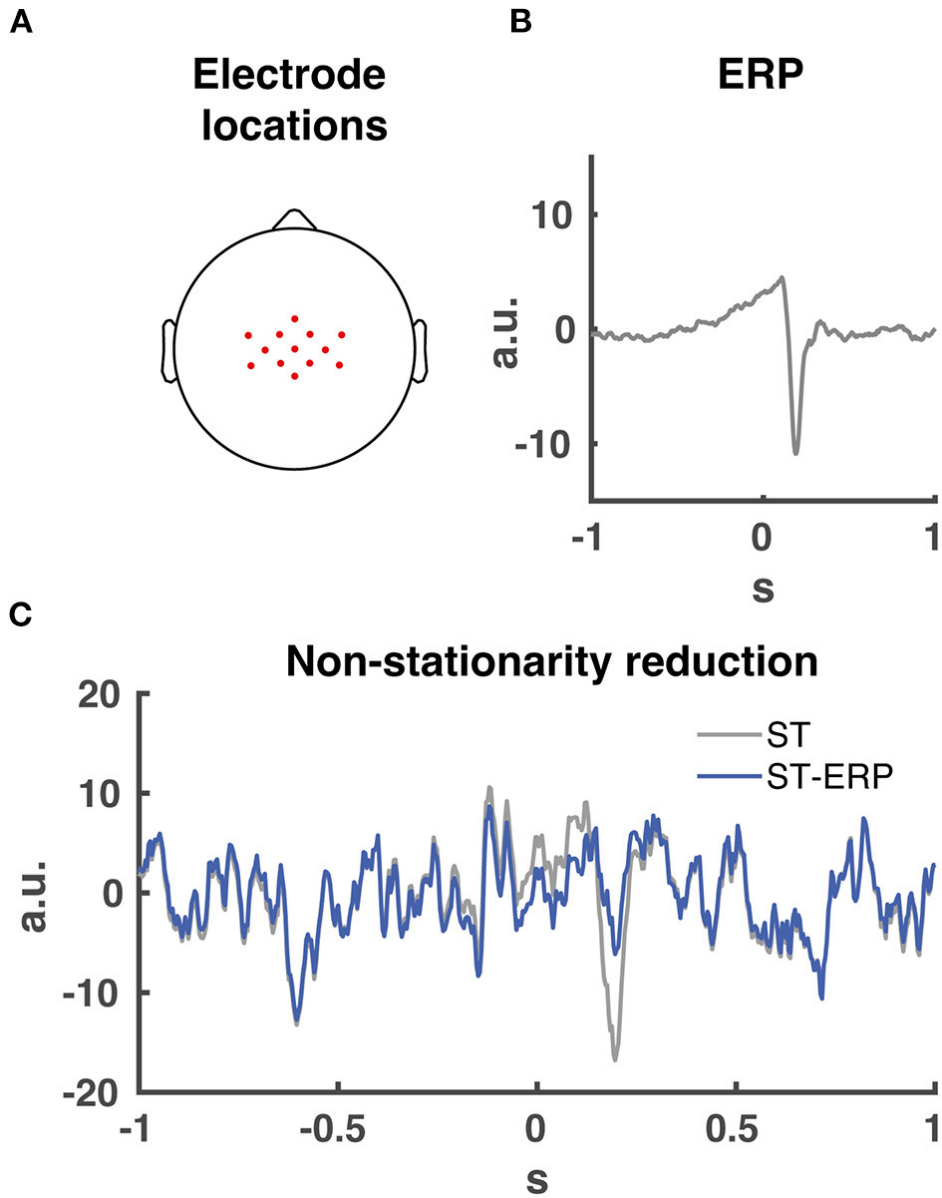
EEG electrode locations and ERP signal removal. **(A)** 13 midfrontal electrode scalp locations averaged together for the Granger causality analysis. **(B)** Participant EEG average event related potential (ERP) from midcentral electrode location CZ time-locked to perturbation onset. **(C)** signal non-stationarity reduction. Time series of single trial (ST) EEG data from midcentral electrode location CZ time-locked to perturbation onset indicated in grey. Time series of ERP subtraction from single trial time series data (ST-ERP) in blue.

Prior to Granger causality computation, we downsampled and normalized (z-score) the EMG activity. Then we subtracted the ensemble average EMG from the single-trial EMG data per participant and muscle. This procedure is necessary for Granger causality analyses involving event related potential to facilitate model-fitting and reduce non-stationarities [(26); see Figure 3C for an example on removing non-stationarities from EEG data].

### 2.4. EEG processing

For the preprocessing of EEG and EOG data, MATLAB functions of the EEGLAB toolbox were used (27). Continuous data were epoched into intervals of −2 to +3 s relative to perturbation onset. Data were bandpass filtered using the ‘filtfilt.m' function (2–200 Hz, consecutive high-pass and low-pass 5th order Butterworth IIR filters, zero-phase shift) and common average re-referenced. Noisy channels were flagged for rejection based on a kurtosis >3 and a variance >3 and rejected based on visual inspection. In addition, epochs were visually inspected for noise. Independent components analysis (Infomax ICA) with a minimum of 90 and maximum of 125 principal components (depending on the rank of the EEG data) was run, and independent components were rejected based on being excessively noisy and of non-brain origin (mean = 91, sd = 16 rejected components; two data sets showed excessive noise resulting in a large mean). Back-projection of the retained independent components resulted in artifact-reduced EEG data. Noise rejected channels were interpolated.

Our and other studies indicated that midfrontal cortical activity plays a major role during the initial phase of the balance response regarding the monitoring and cortical control of the balance response (2, 12, 17, 22). In addition, cortical (pre-)motor regions are located around the midfrontal head location. Therefore we chose a substantial amount of midrontal electrodes, resulting in the 13 selected electrodes (FCz, Cz, CPz, C1, C2, CCP3h, CCP1h, CCP2h, CCP4h, FCC3h, FCC1h, FCC2h and FCC4h; see Figure 3A for topographical locations) centered over the midfrontal scalp location for CMC analysis. Prior to the Granger causality computation, the ERP (Figure 3B) was subtracted from the single-trial data for both EMG and EEG. This process maintains spectral information of the data and reduces the non-stationarities in the signal (Figure 3C).

### 2.5 Data inclusion

We collected a total of 2,491 trials and rejected 149 trials, based on flat lines and artifacts in EEG and EMG data. Of the remaining 2,342 trials (mean = 130, sd = 19 trials per subject), 1,177 were forward and 1,165 were backward perturbations. In the forward direction there were 675 feet-in-place responses vs. 502 step responses. In the backward direction, we recorded 565 feet-in-place responses and 600 step responses.

### 2.6 Granger causality analysis

We applied Granger causality analysis to compute the directional coupling from the cerebral cortex to the muscles between individual EEG channels and EMG data over 1 to 100 Hz with a resolution of 0.05 Hz. For the analysis we used the spectral Granger causality Matlab MVGC toolbox (28, 29). We applied a sliding window of 400 ms to predict EMG activity from the EEG signal (using a smaller window would collapse the frequency interpretation of lower frequencies, whereas a larger window would diminish temporal accuracy). We used a model order of 100 ms, meaning we predicted muscle activity up to 100 ms ahead of the current EEG sample. This 100 ms model order was visually determined through time domain Granger causality tests of multiple participants, identifying a clear Granger causality increase relative to baseline (−1.4 to −0.75 s relative to perturbation onset) and a stationary baseline Granger causality. The time domain Granger causality analysis determined the Granger gain of cortical interaction averaged over all muscles. According to these parameters, Granger causality temporal data should be interpreted as a prediction of EMG from EEG over the past 400 ms window where EEG signal of sample *t*_0_ predicts EMG activity up to sample *t*_100_ (up to 100 ms ahead).

Granger Causality analysis benefits from large amount of data. To optimize the Granger causality outcome we conducted the analysis over a time series of 4 s with a consistent amount of data for all frequency bands and individual muscles. Second, we included all step trials per participant, which resulted in a substantial amount of 500 (forward) and 600 (backward) trials (see 2.5 Data inclusion). In addition, we removed the ERP from both the EMG and EEG data to improve the signal stationarity (see 2.4 EEG processing). The sliding window approach of the Granger causality analysis provided by the MVGC toolbox (28, 29) also improves analysis of non-stationary signals. The sliding window was sufficiently large enough to interpret low frequency (3–8 Hz) data similar to the Peterson study (17). Lastly, the data was averaged over 13 midfrontal electrodes (see 2.4 EEG processing and Figure 3C), which further increased the midfrontal Granger causality signal to noise ratio.

We conducted spectral Granger causality relative to three time-locking events to get a good alignment with the EMG activity. Due to the temporal sliding window of the Granger causality analysis, between-signal comparisons can only be made at specific time locked events. Therefore, we time-locked to three specific events at which we can temporally accurately compare EMG with Granger causality at time 0 s. First, perturbation onset time-locked Granger causality analysis was done to investigate coupling with muscles immediately after perturbation. Secondly, Granger causality time-locked to foot-off event was computed to investigate CMC prior to step initiation and during stepping response. Finally, Granger causality time-locked to foot strike was done to investigate CMC prior to and after foot landing. For each time-locking event we averaged the Granger causality data over all 13 EEG electrodes, meaning that we analyzed one average cortical Granger causality time-frequency map for each muscle.

### 2.7. Statistical analysis

For EMG and CMC time series data, statistical tests were done over all time windows of the time-locked events. Interquartile range latencies were used to determine the upper temporal boundaries for statistical testing. Participant average foot off event latencies did not significantly differ between directions (Ranksum = 391, Z = 1.8, *p* = 0.07; median_*forward*_ = 451 ms, IQR_*forward*_ = 210 ms, median_backward_ = 400 ms, IQR_*backward*_ =202 ms). In addition, foot strikes latencies did not differ for step direction (Ranksum= 362, Z=0.9, p=0.23; median_*forward*_ = 641 ms, IQR_*forward*_ = 214 ms, median_backward_ = 629 ms, IQR_*backward*_ =225 ms). For convenience, we rounded the 75th percentile values up to foot off = 600 ms, foot strike = 800 ms. This resulted in the following analysis time windows; perturbation onset to 600 ms, −200 to 250 ms relative to foot off and−250 to 200 ms relative to foot strike. Please note that there is temporal overlap between these time windows. To determine whether significant EMG activity and CMC occurred relative to baseline activity in response to balance perturbations, multiple sample-wise *t*-tests were conducted time-locked to perturbation onset for each muscle per step and stance leg separately. Given the multiple tests computed in the time domain, the corresponding *p*-values were corrected for false discovery rate [FDR; (30)]. Statistical significance was assessed for critical α = 0.05. For EMG activity, an activity duration threshold of 100 ms and mean baseline activity +1SD (−500 to 0 ms relative to perturbation onset) was used to eliminate premature false positive significance results.

To determine differences between step and stance leg, significant differences between muscle specific step and stance leg EMG activity were computed using a sample-wise *t*-test. Differences in Granger gain for muscle-specific step and stance legs were computed with sample-wise *t*-tests (*p* < 0.05, FDR correction). Temporal windows for significance testing were determined by foot-off and foot strike latencies.

## 3. Results

### 3.1. Corticomuscular coupling

**Figure 4:**
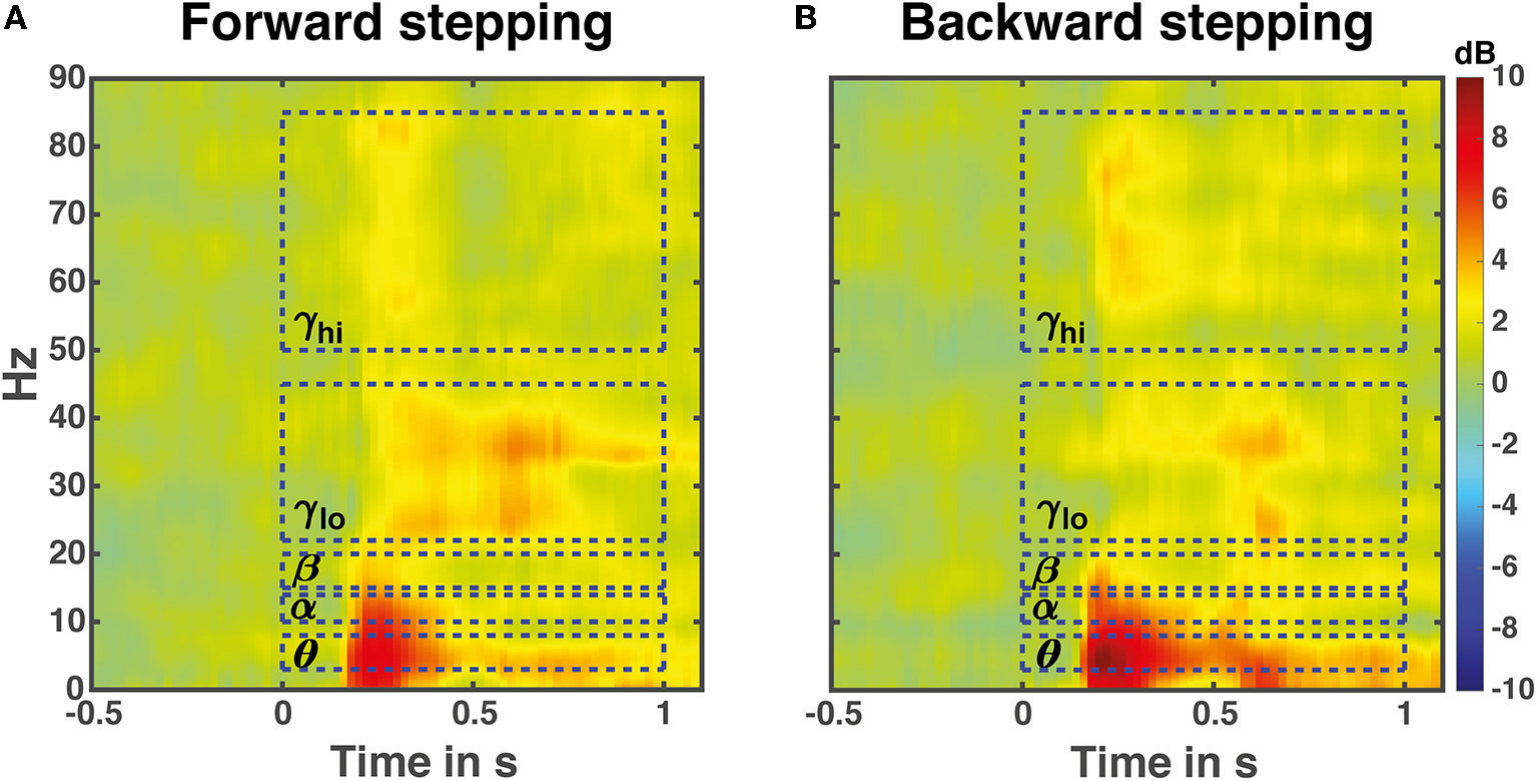
Perturbation time-locked time frequency granger gain. Illustrated data are averaged over all leg muscles in either the forward **(A)** or backward **(B)** stepping direction. The dashed boxes indicate frequency bands of interest for the time course analyses. Note that the temporal boundaries are illustrative; the actual time windows for analysis differed based on stepping times and time-locking events.

Time-frequency analysis averaged over all 13 midfrontal EEG electrodes and muscles in the forward and backward direction revealed increases in Granger causality over multiple frequency bands in response to perturbation onset (Figure 4). The frequencies of interest for further analysis were in the theta (θ: 3–8 Hz), alpha (α: 10–13 Hz), beta (β: 15–22 Hz), low gamma (γ_lo_: 25–40 Hz) and high gamma (γ_hi_: 50–85 Hz) ranges, and are indicated with dashed boxes.

### 3.2. EMG envelopes and frequency-specific CMC

Overall, EMG activity significantly increased compared to baseline in both perturbation directions (forward average EMG onset latency = 152 ms, SD = 37 ms; backward average EMG onset latency = 145 ms, SD = 30 ms; seen in Table 1), indicating that all leg muscles were actively engaged during the stepping responses. All muscles exhibited significant increases in CMC relative to baseline during the reactive step task, though the specific frequency band dynamics varied over time-locking events and muscles (forward average CMC onset latency = 186 ms, SD = 85 ms; backward average CMC onset latency = 171 ms, SD = 83 ms; seen in Table 1). In addition, the onset of significant CMC dynamics following perturbation onset lagged the transient increase in EMG activity. Note that the end of the perturbation onset time window may include some foot-off-related activity. The next sections separately describe for both perturbation directions whether a muscle functions as agonist or antagonist during the specific time locked events. In addition, the next section reports simultaneous muscle frequency specific CMC dynamics per time locked event. For an illustrative summary of the EMG and CMC data and observed significance values, please see the Supplementary Figures S1, S2.

**Table 1:** Onset of significant EMG and CMC dynamics following perturbation onset relative to baseline. Presented data are in ms.

**Forward muscles**												
	**SO**		**TA**		**ST**		**BF**		**RF**			
	**Step**	**Stance**	**Step**	**Stance**	**Step**	**Stance**	**Step**	**Stance**	**Step**	**Stance**	**M**	**SD**
EMG	112	116	152	160	144	144	172	176	184	172	152	26
**CMC**												
Theta	160	180	160	180	160	180	220	200	280	180	190	37
Alpha	180	80		180	220	200	160		260	200	185	52
Beta	160	180	180		20					260	160	87
Low gamma	300				200		0	260	260		204	119
High gamma	0	160	180	220		300		240		240	191	96
								**CMC onset**			186	78
**Backward muscles**												
	**SO**		**TA**		**ST**		**BF**		**RF**			
	**Step**	**Stance**	**Step**	**Stance**	**Step**	**Stance**	**Step**	**Stance**	**Step**	**Stance**	**M**	**SD**
EMG	164	184	96	100	176	156	148	156	136	136	145	30
**CMC**												
Theta	180	180	160	160	220	180	240	160		180	184	30
Alpha						240				220	207	57
Beta		180									180	
Low gamma		80	200	200	340		0	140			163	118
High gamma	100	0	120	200			320	220		140	157	111
								**CMC onset**			178	79

### 3.3. Forward reactive stepping

**Figure 5:**
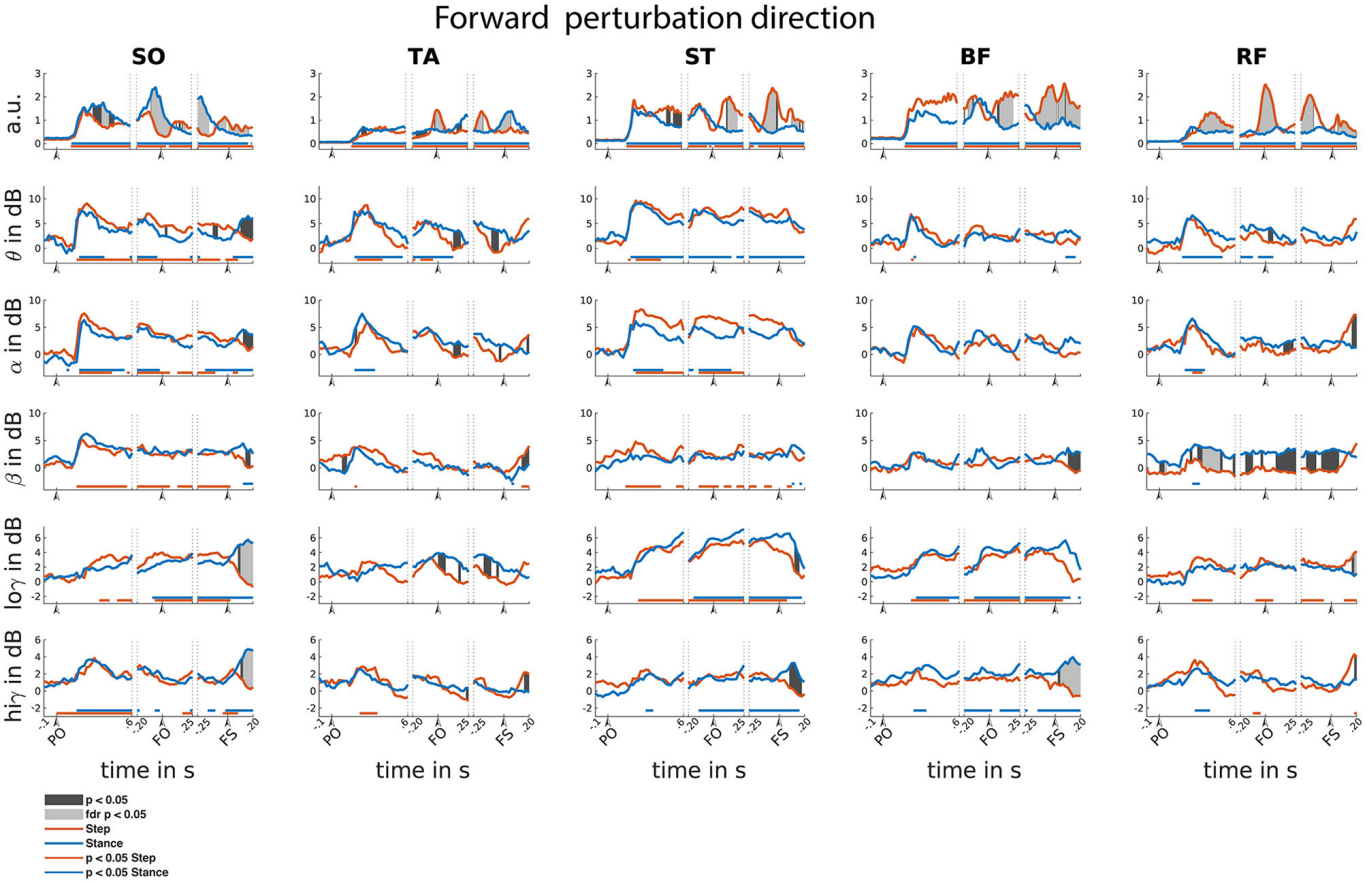
Forward reactive stepping muscle specific EMG and frequency specific CMC. Top row columns contain normalized EMG muscle activity, below are frequency-specific CMC dynamics. Figure columns are leg muscles Soleus (SO), Tibialis anterior (TA), Semitendinosus (ST), Biceps Femoris (BF), Rectus Femoris (RF). Statistically significant differences relative to baseline are indicated using horizontal lines, and differences between step and stance are indicated with grey shaded patches between the time courses. Note that there is temporal overlap between the different temporal windows of interest. Perturbation onset (PO), Foot off (FO), Foot Strike (FS). Foot off latency: Median = 451 ms, IQR = 210ms. Foot strike latency: median = 641 ms, IQR = 214 ms.

Time-locked to perturbation onset, all muscles showed bilateral increase of EMG activity in the initial 150 ms following perturbation onset as steps have not yet been initiated (i.e., both step and stance leg showed similar increased activity; Figure 5). A similar significant increase of CMC was observed across different muscles and over different frequencies. In the theta range, CMC increase was observed in all muscles. Alpha band CMC dynamics were most abundant in both SO,TA,ST and RF muscles, though not significant for the step leg TA. In addition, we observed symmetrically increased beta coupling for all but RF muscles (i.e., similar CMC dynamics between step and stance leg). In the low-gamma frequency band, a gradually and symmetrically increased CMC was observable in the ST and BF muscles. In the high-gamma band CMC increased symmetrically but not significantly across muscles.

Time-locked to foot off, all muscles showed increased EMG activity relative to baseline. Prior to foot off, symmetric EMG activity was observed in both ST and BF muscles. Asymmetric activity was observed in SO (larger in stance leg), TA and RF (larger in stepping leg), consistent with their differential roles in the stance and stepping leg. Observed CMC dynamics did not follow similar patterns to EMG activity within time locked events. Theta dynamics were mainly increased for stance leg muscles and the lower leg muscles of the step leg. Alpha band CMC only increased bilaterally in the SO and ST muscles. In the beta frequency, increased CMC compared to baseline was observed in the step leg SO and ST. Increased CMC in the low-gamma frequency band is observed around the foot off event for all but the TA muscles. The high-gamma band dynamics are overall symmetrical for all muscles and mostly not significantly increased relative to baseline.

Time-locked to the foot strike event, all muscles showed elevated muscle activity relative to baseline. We observed greater EMG activity in step leg TA compared to the stance-leg TA muscle and the other stance leg muscles show increased EMG activity relative to the step-leg.

Although significant CMC dynamics are observed, these did not follow similar activity patterns from the EMG activity. Significant increase in theta CMC was observed prior to foot strike in the step-leg SO and following foot strike event in the stance-leg ST and BF. Alpha band CMC dynamics were limited to both SO muscles. In the beta frequency, step-leg SO and both ST leg muscles showed increased CMC prior to foot strike. In addition, all but the BF muscles showed asymmetrical beta CMC increase following the foot strike event. Low-gamma CMC dynamics were mostly increased symmetrically prior to foot strike in the posterior leg muscles. In addition, both low and high gamma showed asymmetric activation patterns for all except the TA muscles following foot strike.

### 3.4. Backward reactive stepping

**Figure 6:**
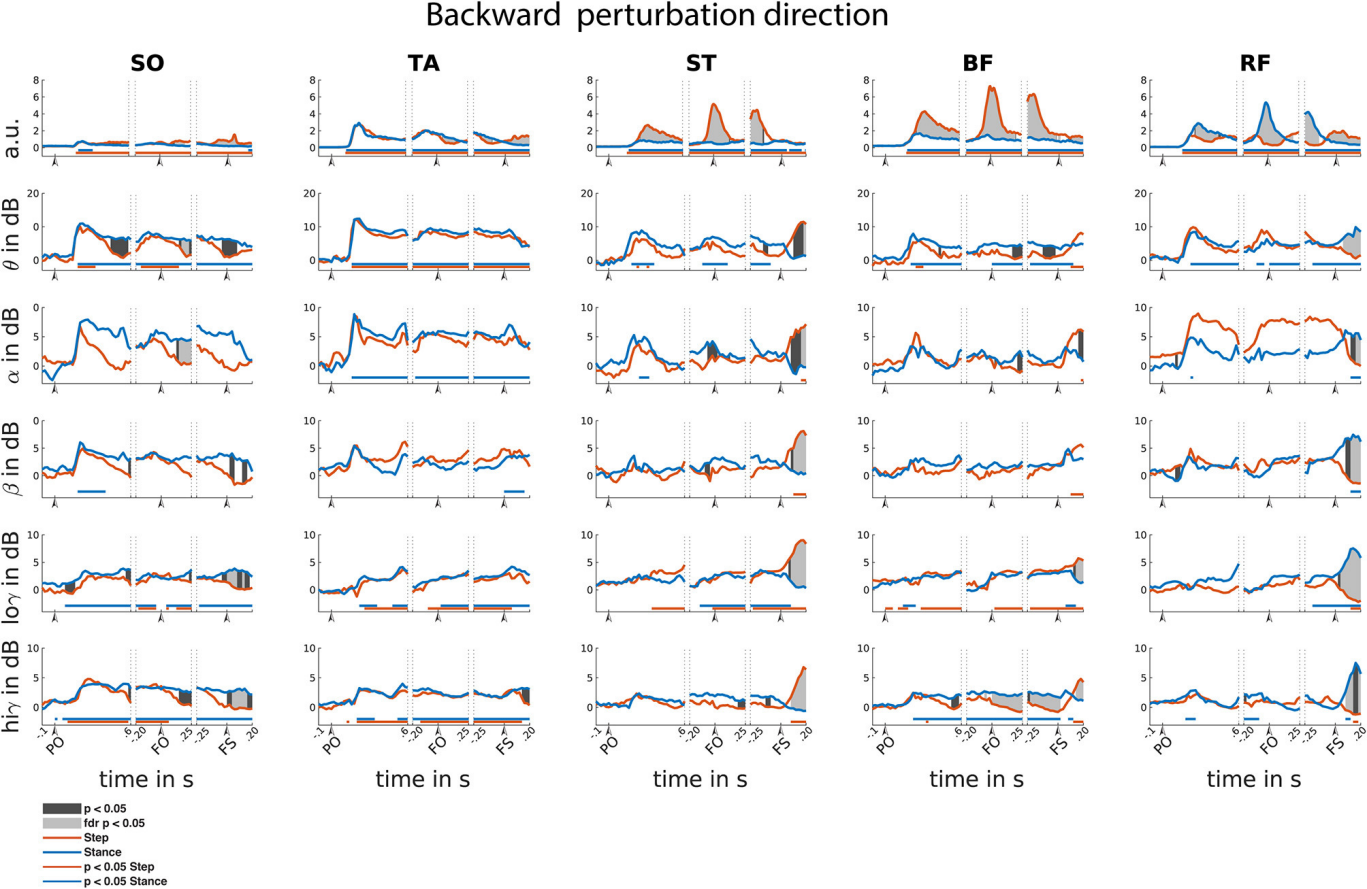
Backward reactive stepping muscle specific EMG and frequency specific CMC. Top row columns contain normalized EMG muscle activity, below are frequency band specific CMC dynamics. Figure columns are leg muscles Soleus (SO), Tibialis anterior (TA), Semitendinosus (ST), Biceps Femoris (BF), Rectus Femoris (RF). Differences relative to baseline are indicated using horizontal lines, and differences between step and stance are indicated with grey shaded patches between the time courses. Note that there is temporal overlap between the different time-locking events. Perturbation onset (PO), Foot off (FO), Foot Strike (FS). Foot off latency: median = 400 ms, IQR = 202 ms. Foot strike latency: median = 629 ms, IQR = 225 ms.

In the backward perturbation direction, EMG activity of all muscles was increased relative to baseline, yet only modestly so in SO. This increased EMG activity is sustained throughout all time-locked events (Figure 6).

Time-locked to perturbation onset, EMG activity transiently and symmetrically increased for all muscles over the initial 200 ms. Similar transient and symmetrical CMC dynamics are observed in the theta range for all muscles. Only stance leg ST and RF showed significant Alpha CMC increase following perturbation onset. Beta CMC dynamics followed a corresponding transient and symmetrical pattern in the lower leg muscles which only resulted in a significant increase for the stance-leg SO. Gradual and modest increase of low-gamma dynamics are observed in posterior leg muscles, albeit not always reaching significance. High-gamma band dynamics overall show similar symmetrical transient cortical interaction patterns as the theta band in all muscles.

Time-locked to the foot off event, all muscles showed increased EMG activity relative to baseline. Prior to foot off, a symmetrical increase in muscle activity was observed in all lower leg muscles. However, upper leg muscles show greater EMG activity in the step leg hamstring and stance leg RF muscles, in line with their agonist roles in backward step initiation. Overall, CMC theta dynamics follow similar symmetrical patterns for the lower leg muscles in comparison to the EMG activity. Yet, dissimilar theta band CMC dynamics compared to EMG were observed in the upper leg muscles. Only increased alpha CMC was observed in the stance leg TA. Please note that the alpha CMC seems elevated for the RF throughout the foot off event while a significant effect remains absent. However, this is caused by strongly increased CMC in one participant specific for this muscle and frequency band. Removal of this participant's data did not influence the observation of additional significant difference effects. Little significant CMC dynamics were observed in the beta band. In the low-gamma band, almost all leg muscles, except for the RF, showed a gradual increase in CMC activity. In the high-gamma frequency band, all lower leg muscles show similar CMC patterns compared to the EMG activity. However, the upper leg muscles overall show little dynamics in comparison to the EMG activity during foot-off event with only slight high-gamma band CMC increases over stance-leg RF and BF muscles.

Time-locked to the foot strike event, all muscles show increased EMG activity and step leg muscles show greater muscle activation. In addition, all muscles showed CMC over multiple frequency bands, yet the observed asymmetrical EMG activation patterns are not mirrored in the CMC patterns in the various frequency bands. All stance leg muscles except the ST muscle show increased CMC in the theta frequency. In addition, the step leg ST and BF showed increased theta CMC following foot strike. Alpha band CMC dynamics were limited to the stance leg SO and the upper leg muscles that showed increased EMG activity prior to the foot strike event. CMC dynamics in the beta band were primarily observed following foot strike for the upper leg muscles that showed increased EMG activity prior to foot strike. Overall, relatively similar CMC dynamics were observed over both the low and high gamma bands.

### 3.5. Differences between step and stance leg

Overall, asymmetrical activation patterns in EMG and CMC between step and stance leg were observed time locked to foot off and foot strike events in both stepping directions. Following a brief symmetrical recruitment of the various muscles shortly after perturbation onset (consistent with an automatic postural response), the activation patterns of stance- and stepping leg muscles started to differ depending on their differential functional roles. Time locked to foot strike, all muscles show difference in CMC activity primarily over beta and gamma frequency bands. Only the RF muscles show similar asymmetrical activity over beta and gamma frequencies compared to the EMG activity following foot strike.

In the backward perturbation direction (Figure 6), the lower leg muscles showed significant EMG activity increases in the step leg during the foot strike time-locked phase of the step response. Whereas, in the upper leg muscles the posterior step leg muscles and stance leg anterior muscles showed consistent increases throughout all three time-locked events of the step response. Prior to foot strike time-locked event, EMG activity in anterior stance leg muscles was lower compared to the step leg EMG activity. Yet following foot strike, EMG activity in these anterior step leg muscles increases relative to the stance leg. Following foot strike, all but the TA muscle show greater EMG activity in the step leg. With respect to the CMC, all but the TA muscle showed distinct differences over all frequency bands following the foot strike event.

## 4. Discussion

Our aim was to identify cortical interactions with various leg muscles through spectral Granger causality during a reactive balance task. We hypothesized that CMC would occur during reactive stepping responses, indicating the contributions of the cerebral cortex to the execution of these responses. In line with our hypothesis, our results illustrated significant CMC increase relative to baseline over multiple muscles and frequency bands following balance perturbations in both directions. In addition, we expected CMC dynamics to differ between step and stance leg. In particular, we expected that stronger CMC would be most evident for the agonist muscles involved in generating the stepping movements, and that an increase in CMC would precede an upregulation of EMG activity in these muscles. Contrary to our expectations, our data illustrated that step and stance leg CMC generally did not show similarity with EMG data (i.e., increases in CMC did not align with increases in EMG activity). Our findings shed new light on cortical involvement of dynamic postural responses with implications for future studies involving clinical populations with deficient CMC (such as stroke).

### 4.1. Increase in CMC during reactive balance stepping

Granger causality analysis during reactive step responses revealed cortical interaction over multiple frequency bands for all muscles in both stepping directions, showing that on average the cortex becomes actively involved in the execution of reactive stepping response at ~180 ms post perturbation. Overall, we observed significant CMC increases in the theta, beta and both gamma frequency bands relative to baseline for individual muscles in either perturbation direction and throughout the three time locked events. In the backward direction, notable broadband interaction was present in upper leg muscles following the foot strike event. Although such broadband CMC following foot strike may suggest that observed dynamics were caused by an artifact, increased broadband CMC in the stance leg muscles during forward stepping and the absence of similar broadband CMC in other step leg muscles (the leg that receives most movement impact that may cause artifacts) support the interpretation of a causal cortical interaction that spanned multiple canonical frequency bands. We propose that the broadband interaction with specific muscles may emphasize the importance of these respective muscles in maintaining a stable posture following the stepping response and we will elaborate on our arguments below. As multiple studies investigated distinct functional roles of cortical rhythms, we will separately discuss their potential roles during the reactive stepping response.

#### 4.1.1. Theta band coupling

Strong transient cortico-muscular interactions in the theta frequency band were observed following perturbation onset in both perturbation directions for almost all leg muscles, indicating that theta dynamics may play a general role in the initial reactive stepping response. Interestingly, after the foot strike event in the backward stepping direction CMC in the theta frequency band mostly involved the muscles that showed increased muscle activity around foot off (i.e., stance-leg RF and step-leg BF), suggesting that the leg muscles that primarily showed increased EMG activation also require relatively more cortical interaction in the later phase of the response. Our findings are in agreement with an increase in CMC in the theta frequency band observed in lateral pull perturbations while standing, which was speculated to facilitate muscle recruitment of the feet-in-place balance response (17). Thus, the increase of CMC in the theta frequency band in the current study may emphasize the importance of the theta rhythm in muscle control following the perturbation onset and termination of the stepping response.

#### 4.1.2. Alpha band coupling

Increased dynamics in the alpha band has been coupled to sensorimotor processes and motor readiness, and it may facilitate a similar role during the reactive balance response. Several studies reported increased alpha coupling during standing compared to walking (17, 31, 32). Although not all muscles showed increased CMC in the alpha band, most dynamics were primarily observed following perturbation onset and time locked to foot off event. These events require specific muscle activation to initially respond to the balance perturbation and initiate the stepping response. Therefore, our results may hint that the alpha frequency signals for muscle readiness in the reactive balance response.

#### 4.1.3. Beta band coupling

The cortical beta frequency band is known for its close relation with voluntary muscle recruitment in a wide variety of tasks (33) and we reasoned that it may play a similar role during the muscle recruitment to facilitate the reactive stepping response. Although relatively few muscles showed significant beta coupling, beta CMC dynamics were most evident following perturbation onset and after foot strike. During these phases, muscle activity was elevated compared to baseline and yet relatively little changes in EMG dynamics occurred, suggesting a rather isometric (semi-static) muscle contraction. Previous studies investigated isometric leg muscle contractions, observing increased CMC in the beta frequency band (14, 34, 35), suggesting that beta dynamics play a role in leg muscle control. In addition, CMC in the beta band has been demonstrated during quiet standing postural control (36) and during feet-in-place balance responses following small lateral perturbations in unipedal stance (21). Interestingly, *increase* in beta band CMC is linked to increase in muscle activity, while a simultaneous power *decrease* of the cortical beta rhythm (note: cortical beta power should not be confused with cortical beta coupling) is observed during sustained muscle contraction (37). Yet, although a decrease in beta power has been reported following perturbation onset for either feet-in-place and step response (2, 12, 17, 22), no consistent increase in beta CMC dynamics was observed in the present study and that of Peterson (17). As beta CMC has mainly been observed during muscle activity in (semi-)static postures, we speculate that the general lack of significant beta CMC may be due to the dynamic nature of the reactive stepping response, with beta CMC becoming somewhat more evident when participants maintained a (semi-)static posture following foot strike. Therefore, we propose that the observed beta CMC in our study may relate to relatively isometric muscle recruitment facilitating stability in this phase of the reactive step response.

#### 4.1.4. Gamma band coupling

Gamma coupling dynamics scale with increased muscle contraction and coordination (14, 38), and may facilitate a similar increased muscle recruiting role during reactive balance stepping. The cortical gamma rhythm covers a wide frequency range and although we analyzed two distinct frequency bands, cortical coupling in either band occurred mostly between foot off and foot strike. Therefore, we will discuss these results as general gamma dynamics [for an extensive review on gamma oscillations and CMC during the control of movements see Ulloa (38)]. Interestingly, we observed gamma rather than beta coupling throughout the reactive step response. Relative increase over CMC dynamics in the gamma band compared to beta band coupling has been linked to an increase in force and muscle coordination during isotonic compared to isometric muscle contraction of ankle and knee joints (14). Therefore, we speculate that the greater need for coordinated muscle recruitment during the step response may result in greater gamma CMC dynamics.

### 4.2. No evidence that lateralized EMG activity is driven by lateralized cortical coupling

Distinct cortical interactions with step or stance leg mainly occurred with respect to the foot off and foot strike events in either direction, suggesting that although the cortex was initially engaged following perturbation onset (as evidenced by the increased coupling relative to baseline; see Figures 5, 6), surprisingly little leg-specific interactions were observed under the hypothesis that transcortical loops are involved in facilitating the reactive stepping response (20, 39). Interestingly, distinct CMC between step and stance leg in both stepping directions were also not mirrored in similar asymmetric EMG activity, contrasting with our expectation that distinct cortical interaction would precede distinct increase in EMG activity. Previous studies did find a correlation between CMC and EMG (13, 15, 34), but these studies concerned voluntary movements and thus involved intentional top-down regulation of muscle activity. In contrast, the stepping responses in the present study were executed in response to an unexpected balance perturbation and were thus *reactive* in nature. We speculate that the difference between feedforward and feedback control may explain these discrepant findings regarding the relationships between CMC and EMG patterns. While the initial phase of the balance recovery response (i.e., automatic postural response) is known to be mediated by subcortical circuits (20), the present study may hint at these circuits also playing a greater-than-expected role during leg specific muscle activations of the stepping response.

### 4.3. Clinical implications

Our results demonstrate CMC throughout the reactive stepping response, indicating that impairments in any of the cortico-muscular communication circuitry may lead to dysfunctional cortico-muscular interaction underlying the impaired balance response. Indeed, impaired stepping responses have been reported for a variety of conditions of the central nervous system (e.g., stroke and Parkinson's disease (40, 41); which may in turn contribute to their elevated risk of falling (42, 43). Yet, the specific mechanisms underlying reactive stepping impairments are still poorly understood. Our work indicates that the application of Granger causality analysis for studying muscle and frequency specific deviations may help provide valuable insights in the underlying pathophysiology of impaired balance response in these clinical populations.

### 4.4. Limitations and strengths

The current study is exploratory and there are some limitations to consider. An important limitation is the sliding window approach used in the Granger causality analysis, meaning that observed effects are smeared over time. Yet, while peak activity is not systematically biased by the windowing, temporal comparison between EMG and CMC timeseries needs to be done with care, as events time locked to foot-off and foot strike contain a signal mixture related to other events due to the large sliding window and the heterogeneity of response latencies.

The experiment involved balance perturbations of different intensities resulting in stepping responses. As perturbation intensity is known to affect the amplitude of the perturbation evoked potential, the ERP contains the average of all perturbation intensities (i.e., low and high intensities and thus small and large amplitudes). While subtracting the ERP from the single trial data, we may have induced minor non-stationarities. Therefore, we recommend future studies to include perturbation intensities of a single intensity that is strong enough to elicit step responses.

On the other hand, our study shows novelty and strengths in several aspects. First of all, CMC at key events throughout the stepping response elicited temporal evolution of distinct spectral dynamics with respect to EMG. Future studies may further investigate the specific role of the spectral dynamics with respective muscles throughout the balance response. In specific, it is of interest to investigate the role of each frequency band with respect to the functional muscular role during the reactive step response. In addition, the current study is the first to conduct separate analysis of step- and stance-leg CMC in comparison to the temporal evolution of EMG activity. The surprising dissimilarity that we observed in CMC and EMG patterns between the legs indicates that the cortex does not appear to facilitate EMG recruitment for executing the balance recovery step in a refined leg- and muscle specific manner.

## 5. Conclusion

We conclude that reactive balance responses require direct interactions from the cortex with the individual muscles, yet without substantial leg-specific differences in CMC patterns. Our work is relevant for clinical populations with impaired balance control, where CMC analysis may elucidate the underlying pathophysiological mechanisms.

## Data availability statement

The original contributions presented in the study are publicly available. This data can be found here: The Donders Repository (https://data.donders.ru.nl/) via di.dccn.DSC_4220000.06_843, ‘EEG of human balance control'.

## Ethics statement

The studies involving human participants were reviewed and approved by Research Ethics Committee of the Radboud University Medical Center (Nijmegen, The Netherlands; Dossier 2018-4970). The patients/participants provided their written informed consent to participate in this study.

## Author contributions

MS: designed research, analyzed data, and wrote paper. TS-E, MC, and VW: supervised MS throughout the study and provided feedback. All authors contributed to the article and approved the submitted version.
