# Supplementary material for: Distinct cortico-muscular coupling between step and stance leg during reactive stepping responses
Source: Front Neurol. 2023 Mar 14;14:1124773. doi: 10.3389/fneur.2023.1124773 (PMC10043329; doi:10.3389/fneur.2023.1124773)
Supplement: Supplementary file 1 [file Data_Sheet_1.docx]

To summarize our time series EMG and CMC observations, we provide the data averaged over time. For this figure we took the following ROI’s per event. 100 - 300ms for the Perturbation onset time window. 0 - 200ms for both the foot off and foot strike events.

Figure S1
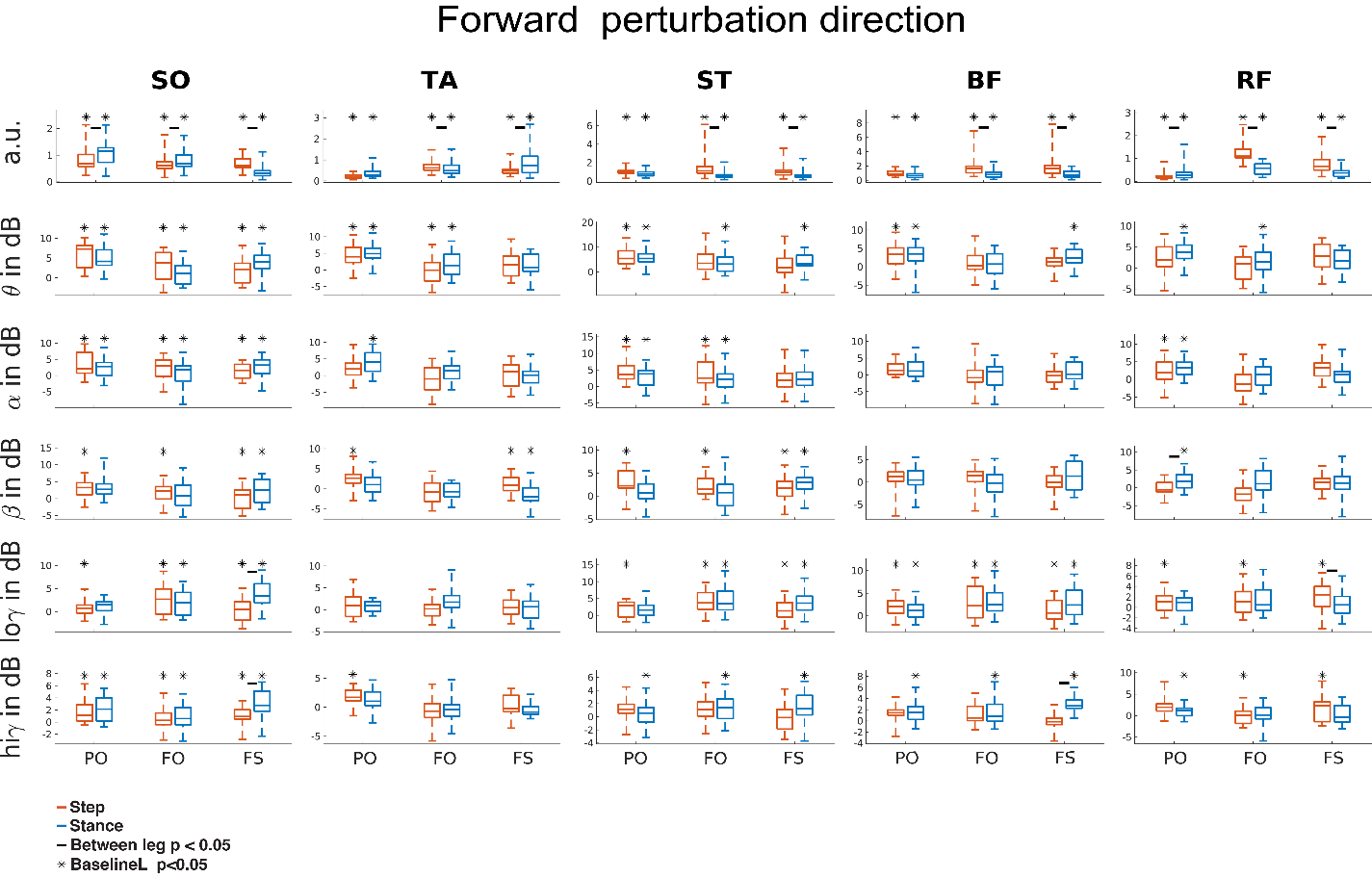


Figure S1. Top row columns contain normalized EMG muscle activity, below are frequency-specific CMC dynamics. Figure columns are leg muscles Soleus (SO), Tibialis anterior (TA), Semitendinosus (ST) , Biceps Femoris (BF), Rectus Femoris (RF). Statistically significant differences relative to baseline are indicated using an asterisk, and differences between step and stance are indicated with a horizontal dash. Perturbation onset (PO), Foot off (FO), Foot Strike (FS)

Figure S2


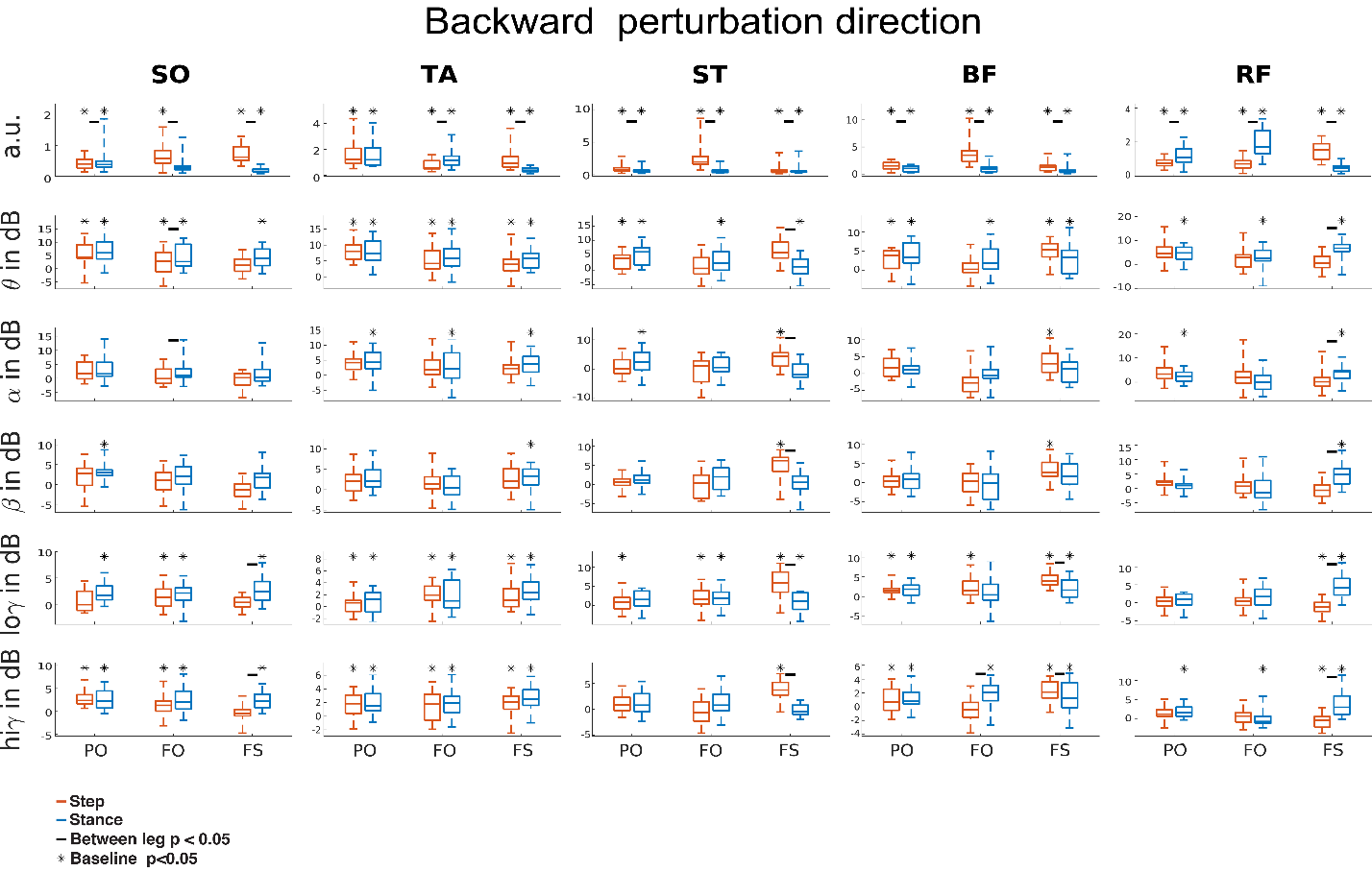


Figure S1. Top row columns contain normalized EMG muscle activity, below are frequency-specific CMC dynamics. Figure columns are leg muscles Soleus (SO), Tibialis anterior (TA), Semitendinosus (ST) , Biceps Femoris (BF), Rectus Femoris (RF). Statistically significant differences relative to baseline are indicated using an asterisk, and differences between step and stance are indicated with a horizontal dash. Perturbation onset (PO), Foot off (FO), Foot Strike (FS)
